# The classification method of donkey breeds based on SNPs data and machine learning

**DOI:** 10.3389/fgene.2025.1496246

**Published:** 2025-04-09

**Authors:** Dekui Li, Xiaolong Hu, Yongdong Peng

**Affiliations:** ^1^ Department of Computer Science, Hubei Water Resources Technical College, Wuhan, China; ^2^ School of Computer Science, Liaocheng University, Liaocheng, China; ^3^ School of Agricultural Science and Engineering, Liaocheng University, Liaocheng, China

**Keywords:** donkey breed classification, SNPs, SMOTE, machine learning, LOOCV

## Abstract

A method for accurately classifying donkey breeds has been developed by integrating single nucleotide polymorphism (SNPs) data with machine learning algorithms. The approach includes preprocessing donkey genomic sequencing data, addressing data imbalance with the Synthetic Minority Over-sampling Technique (SMOTE), and utilizing an improved Leave-One-Out Cross-Validation (LOOCV) for dataset partitioning. Support Vector Machine (SVM), K-Nearest Neighbors (KNN), and Random Forest (RF) models were constructed and evaluated. The results demonstrated that different chromosomes significantly influence classifier performance. For instance, chromosome Chr2 showed the highest classification accuracy with KNN, while chromosome Chr19 performed best with SVM and RF models. After enhancing data quality and addressing imbalances, classification performance improved substantially, with accuracy, precision, recall, and F1 score showing increases of up to 15% in certain models, particularly on key chromosomes. This method offers an effective solution for donkey breed classification and provides technical support for the conservation and development of donkey genetic resources.

## 1 Introduction

Donkeys, as an integral part of livestock resources, play a significant role in global biodiversity conservation (Huang et al., 2023). However, with societal advancements, the breeding and utilization of donkeys have sharply declined, leading to a severe threat to the genetic resources of donkey populations, with many breeds now endangered (Seyiti and Kelimu, 2021). The development and conservation of donkey genetic resources face significant challenges (Wang et al., 2022). To address these challenges, it is imperative to explore efficient and accurate methods for classifying donkey breeds, to select superior breeds for breeding and identify endangered breeds for protection (Wang et al., 2020).

Traditional methods for identifying donkey breeds primarily rely on biological experiments and morphological characteristics. However, these methods are time-consuming, labor-intensive, and prone to environmental and conditional influences, leading to lower classification accuracy (Franco-Duarte et al., 2019; Hosseini et al., 2019).

Single Nucleotide Polymorphisms (SNPs) refer to variations at a single nucleotide position in the genome, representing the most common form of genetic variation in the human genome. The study of SNPs dates back to the late 1990s when scientists first recognized the widespread presence of these variations among individuals and their potential in associating genes with traits (Lander, 1996).

SNPs typically occur in populations at a certain frequency, and their widespread distribution across the genome makes them ideal genetic markers (Waterston et al., 2002). With the advent of high-throughput sequencing technologies, the detection of SNPs has become more efficient and cost-effective, significantly advancing SNP-related research. These technologies have enabled researchers to identify millions of SNPs and apply them to various research purposes, including disease association studies, population genetics, evolutionary biology, and breeding research in agricultural sciences (Mccarthy et al., 2004).

In genomic research, the classification of SNPs has become a focal area of study. Traditionally, SNPs classification has relied on biological experiments and morphological characteristics. However, with the development of bioinformatics, machine learning techniques have been widely applied to SNPs classification tasks. Modern SNPs classification methods typically combine high-dimensional SNPs data with various machine learning algorithms to identify genetic variations associated with specific phenotypes, diseases, or drug responses (Qi, 2012).

The advantages of SNPs classification include its ability to process vast amounts of genomic data and automatically identify potentially useful information through computational models, thereby improving research efficiency and accuracy. Additionally, SNP classification helps reveal complex relationships between genes and traits, which has important applications in disease prediction, personalized medicine, and breeding in crops and livestock (Schiavo et al., 2020; Silva et al., 2022).

However, SNPs classification also faces several challenges. First, the high dimensionality and complexity of SNPs data make it difficult for traditional data processing methods to be effectively applied. Second, linkage disequilibrium (LD) between SNPs, where certain SNPs tend to be inherited together, can form LD blocks in specific genomic regions, potentially affecting the accuracy of classification models (Altmüller et al., 2001). Furthermore, due to data quality issues and insufficient sample sizes, SNPs classification may also encounter errors and inaccuracies in practical applications (Whalen et al., 2022).

In recent years, with the development of bioinformatics, the integration of single nucleotide polymorphism (SNPs) data and machine learning has provided a new and efficient approach for classifying donkey breeds (Ho et al., 2019). SNPs, as a third-generation molecular marker technology, significantly impact the expression of traits across different breeds, making them ideal genetic markers for studying phenotypes and diseases (Lander, 1996; Srivastava et al., 2023). Concurrently, machine learning algorithms, known for their efficacy in handling and analyzing high-dimensional data, have been widely applied to the processing of SNPs data (Thottakkara et al., 2016). As research in this area has progressed, the feasibility and effectiveness of combining machine learning with SNPs for classification have been increasingly validated (Ban et al., 2010).

However, traditional SNPs classification methods based on machine learning often rely on large amounts of accurate sample data (Ho et al., 2019). Due to practical limitations and cost factors, obtaining large sample sizes is often challenging (Macgregor et al., 2008). Additionally, during donkey crossbreeding, the expression of certain SNPs may be less pronounced, and the data processing phase may introduce significant errors, making classification in practical applications difficult (Shen et al., 2021). Therefore, developing a method to quickly filter out errors in small sample data is crucial (Silva et al., 2022). Such a method would not only enhance classification accuracy but also reduce model bias caused by data quality issues, thereby providing more reliable technical support for accurate donkey breed classification (Schiavo et al., 2020).

To address these challenges, this study proposes a novel approach that integrates SNPs markers, Synthetic Minority Over-sampling Technique (SMOTE), machine learning algorithms, and an improved Leave-One-Out Cross-Validation (LOOCV) method for the precise classification of donkey breeds. The study utilized unlabelled SNPs data from nine different donkey breeds, applied the improved LOOCV technique for dataset partitioning, and balanced the dataset using SMOTE, aiming to resolve various issues and challenges exposed during data processing. In selecting classification algorithms, the study employed three machine learning models commonly used in genetic data analysis: Support Vector Machine (SVM), K-Nearest Neighbors (KNN), and Random Forest (RF). The results demonstrate that the proposed workflow performs exceptionally well in classifying and predicting donkey breeds, particularly in addressing challenges related to small sample sizes, data errors, and data imbalance. By combining SNPs markers, SMOTE, machine learning algorithms, and an improved LOOCV method, this workflow shows significant advantages in the precise classification of donkey breeds, offering reliable technical support for the rapid identification and conservation of these breeds.

## 2 Methods

### 2.1 Machine learning algorithms

To achieve accurate prediction of donkey breeds, this study employs three classical machine learning methods widely used in genomic data analysis and classification: Support Vector Machine (SVM), K-Nearest Neighbors (KNN), and Random Forest (RF). These methods have proven effective in the processing and classification of SNP data (Qi, 2012; Silva et al., 2022).

Support Vector Machine (SVM) is a widely used supervised learning model whose core idea is to construct an optimal hyperplane for classification, maximizing the margin between different classes. SVM performs well in handling high-dimensional data, particularly by using kernel functions (such as polynomial and radial basis functions) to map data into a higher-dimensional feature space, making originally non-linearly separable data linearly separable in this space. This characteristic makes SVM especially advantageous in processing complex SNPs data (Cortes and Vapnik, 1995). In recent years, SVM has been widely applied in SNPs classification studies. For instance, Ban et al. (2010) used SVM combined with SNPs data to successfully identify gene combinations associated with type 2 diabetes, demonstrating SVM’s strong capabilities in processing high-dimensional genomic data (Ban et al., 2010). Huang et al. (2018) used SVM in their study to successfully identify SNPs combinations associated with cancer susceptibility, further proving SVM’s effectiveness and robustness in high-dimensional genomic data processing (Huang et al., 2018).

K-Nearest Neighbors (KNN) is a commonly used lazy learning algorithm, where classification decisions are based on the distance between a sample point and all samples in the training set. KNN finds the K nearest neighbors and determines the sample’s classification based on the majority vote of these neighbors. The simplicity and intuitiveness of the KNN algorithm make it particularly suitable for small-scale datasets, especially in SNPs data with local structure, where KNN can effectively identify and classify tag SNPs (Yang et al., 2010; Russ, 2023).

Random Forest (RF) is an ensemble learning method that classifies by constructing multiple decision trees based on random subsets. The advantage of RF lies in its excellent generalization performance and resistance to overfitting, particularly when processing high-dimensional data and large datasets (Qi, 2012). Additionally, RF has a built-in feature importance evaluation mechanism that can identify the most critical SNPs in classification tasks (Bertolini et al., 2018; Venkat, 2018).

These three machine learning methods—SVM, KNN, and RF—offer complementary strengths in addressing the challenges associated with SNP-based classification tasks, such as handling high-dimensional data, identifying local structures, and improving generalization performance. By employing these methods in this study, we aim to evaluate their effectiveness in the context of small-sample SNP datasets for donkey breed classification. This comprehensive comparison provides critical insights into the suitability of different algorithms for genomic data analysis and serves as a foundation for developing robust classification frameworks tailored to the unique challenges of SNP datasets in conservation genomics.

### 2.2 Synthetic minority over-sampling technique (SMOTE)

In small sample classification research, handling imbalanced datasets is a key challenge. The Synthetic Minority Over-sampling Technique (SMOTE) is a commonly used method that addresses this issue by generating artificial data points to increase the number of minority class samples, thereby improving the balance of the dataset and enhancing the model’s ability to recognize minority classes (Chawla et al., 2002).

The basic operations of SMOTE involve randomly selecting a sample from the minority class, calculating the distance between this sample and its K nearest neighbors, randomly selecting N samples from these neighbors, and performing random linear interpolation on these samples to generate new minority class samples. Finally, these synthetic samples are combined with the original dataset to form a new, balanced training set. Because this method primarily relies on nearest neighbor calculations and linear interpolation, it can be efficiently applied even in small sample datasets (Blagus and Lusa, 2013).

In the context of SNPs classification, the small sample problem is particularly significant. Due to the high dimensionality and complexity of genomic data, small sample classification often faces challenges such as insufficient sample sizes, class imbalance, and varying data quality. SMOTE technology can alleviate these problems by generating diverse and representative synthetic samples, thereby enhancing the generalization ability and accuracy of classification models (Whalen et al., 2022). In SNPs classification tasks, the use of SMOTE and other over-sampling techniques can significantly improve classifier performance. For example, Bertolini et al. (2018) applied SMOTE to handle imbalanced data in SNPs classification for cattle breeds, using a random forest model for classification. The results showed that this method effectively improved the classification accuracy of minority SNPs (Bertolini et al., 2018). Similarly, Silva et al. (2022) used SMOTE in their research, combined with machine learning algorithms, to enhance the accuracy of SNPs classification under small sample conditions (Silva et al., 2022).

Overall, SMOTE improves the balance of datasets by generating synthetic data, thereby effectively enhancing the classifier’s ability to recognize minority classes in small sample classification. Particularly in SNPs classification, SMOTE technology generates more representative synthetic samples by considering the local structure of the data, significantly improving the overall performance of classifiers.

This study employed the SMOTE to handle imbalanced datasets in small sample classification tasks. In this study, the parameters for SMOTE were set as follows: the value of *k_neighbors* was set to 1, meaning that the nearest sample among the minority class neighbors was selected to generate new synthetic samples. The final number of samples in each class was expanded to 100, ensuring balance across classes during the over-sampling process. The generated synthetic samples were then combined with the original dataset to form a new balanced training set. These parameter settings allowed us to preserve the characteristics of the original data while minimizing the risk of overfitting caused by over-sampling.

### 2.3 Cross-validation techniques

Cross-validation is a widely used technique in machine learning to evaluate the performance and generalization ability of models. It works by partitioning the dataset into multiple subsets, with some used for training and others for validation. By repeating this process across different subsets, cross-validation helps to reduce the risk of overfitting and provides a more reliable estimate of model performance (Kohavi, 1995). Common cross-validation methods include k-fold cross-validation, stratified k-fold cross-validation, and leave-one-out cross-validation (LOOCV).

The primary advantage of cross-validation lies in its ability to provide robust estimates of a model’s predictive performance across unseen data, making it an essential step in model selection and hyperparameter tuning. In k-fold cross-validation, for instance, the dataset is divided into *k* equally sized folds. Each fold is used once as the validation set, while the remaining *k*−1 folds are used for training. This process is repeated *k* times, and the results are averaged to provide a final performance metric (James et al., 2013). Stratified k-fold cross-validation further improves this approach by ensuring that the proportion of samples from different classes in each fold is consistent with the overall dataset, which is particularly important for imbalanced datasets (Wong and Yeh, 2019).

Cross-validation techniques play a critical role in genomic studies, especially those involving SNPs data, due to the high dimensionality and complexity of the datasets. SNPs datasets often contain thousands of features but relatively few samples, creating challenges such as overfitting and data sparsity. Cross-validation helps address these challenges by enabling rigorous model evaluation and selection without the need for additional data collection, which is often costly and time-consuming (Wang et al., 2022).

In SNP classification studies, Leave-One-Out Cross-Validation (LOOCV) has been extensively applied because it allows for the precise assessment of predictive models even with small sample sizes. For instance, LOOCV has been used to evaluate machine learning models in identifying disease-associated SNPs, predicting phenotypic traits, and distinguishing between different breeds in livestock genetics (Schiavo et al., 2020; Silva et al., 2022). Additionally, it helps researchers identify overfitting issues by ensuring that each sample is tested independently, which is crucial for validating models that process high-dimensional genomic data.

In this study, we utilize LOOCV, the specialized form of cross-validation where each sample in the dataset is used once as the validation set while the remaining samples form the training set. LOOCV is especially useful for small sample sizes, as it maximizes the use of available data for both training and validation. While computationally expensive, LOOCV is often regarded as the gold standard for performance evaluation in small datasets because it provides the most exhaustive use of data and reduces bias in performance estimates (Astrologo et al., 2023).

The use of LOOCV in this study aligns with the specific requirements of SNP datasets for donkey breed classification. Given the small sample size and high dimensionality of SNP data, LOOCV provides a thorough evaluation framework to test the generalization ability of models and avoid overfitting. By integrating LOOCV with machine learning algorithms and data balancing methods, this study aims to develop a reliable framework for accurate classification of donkey breeds, contributing to genomic research and conservation efforts.

### 2.4 Performance evaluation

The performance of models is typically evaluated using a series of metrics, including Accuracy, Precision, Recall, and F1 Score. These metrics, derived from the confusion matrix, provide a comprehensive assessment of a model’s performance across different dimensions.

Accuracy: Represents the proportion of correctly predicted samples to the total number of samples, measuring the overall correctness of the model.

$$\text{Accuracy}=\frac{TP+TN}{TP+FP+FN+TN}$$

Precision: Represents the proportion of true positive predictions among all samples predicted as positive, used to measure the model’s prediction accuracy.

$$\text{Precision}=\frac{TP}{TP+FP}$$

Recall: Represents the proportion of true positive samples successfully predicted as positive, used to measure the model’s ability to identify positive cases.

$$\text{Recall}=\frac{TP}{TP+FN}$$

F1 Score: Is the harmonic mean of Precision and Recall, providing a balanced measure of both accuracy and completeness.

$$F1\text{ Score}=2\times \frac{\text{Precision}\times \text{Recall}}{\text{Precision}+\text{Recall}}$$

Where Total Positive (P) represents the number of actual positive samples, and Total Negative (N) represents the number of actual negative samples. The four main terms in the confusion matrix are explained as follows:

True Positive (TP): The number of samples correctly predicted as a specific donkey breed.

True Negative (TN): The number of samples correctly predicted as not belonging to a specific donkey breed.

False Positive (FP): The number of samples incorrectly predicted as a specific donkey breed.

False Negative (FN): The number of samples incorrectly predicted as not belonging to a specific donkey breed.

### 2.5 Experimental design

The experimental design of this study is outlined in Table 1. Data preprocessing is a crucial first step in any machine learning task. In this stage, the collected donkey genomic sequencing data is subjected to cluster analysis and partitioning to lay the foundation for subsequent work. Cluster analysis helps reveal SNPs within the data at the population level and divides the data into subsets with similar characteristics, thereby aiding the machine learning algorithms in better understanding and processing the data. The data is then further organized and divided according to chromosomes, providing more structured input for the classification tasks. This ensures that during the processing of each genome, different chromosomal information is distinguished, thereby enhancing the accuracy and reliability of the classification models.

**TABLE 1:** Algorithm workflow.

**Algorithm:** Construction and Evaluation of Classification Models Based on SNPs Data
**Input:** Data File Set DDD: Each file contains features and class labels of samples. Classifier Set CCC: A set of different classification algorithms (SVM, KNN, RF).
**Output:** Classification performance evaluation results for each data file using different classifiers.
**Process** Initialize Results = [] For each data file d in D: Preprocess d (cluster analysis, organize by chromosomes) Initialize FileResults = (Wong and Yeh, 2019) Partition data using LOOCV Apply SMOTE to handle class imbalance For each classifier clf in C: Initialize Accuracies, All_y_true, All_y_pred For each sample i: Train clf on the training set after SMOTE Predict labels for the test set Store y_test and y_pred Calculate avg_accuracy, precision, recall, F1 score Save evaluation metrics to FileResults Store FileResults in Results Export Results

Following data partitioning, the LOOCV method is used to divide the preprocessed data into training and testing sets. To address the imbalance in the dataset, the Synthetic Minority Over-sampling Technique (SMOTE) is introduced. SMOTE generates synthetic minority class samples, balancing the dataset and improving the model’s ability to recognize minority classes.

The third step involves building the machine learning classification models. In this step, appropriate machine learning algorithms (SVM, KNN, and RF) are selected, and the training set is fed into the models for training. The most suitable model is chosen based on the nature of the task and the characteristics of the data, with continuous training and tuning to achieve optimal classification performance.

Finally, the performance of the established models is evaluated using the test set and various evaluation metrics. This includes using accuracy, precision, recall, and F1 score to assess the model’s performance on the test set. These metrics provide a comprehensive evaluation of the model’s predictive ability and determine whether the model has achieved the desired results. Based on the evaluation outcomes, necessary adjustments and improvements are made to further enhance the classification accuracy and stability of the models.

#### 2.5.1 Data preprocessing

This study analyzed sequencing data from nine distinct donkey breeds, comprising a total of 37 samples. The sample distribution among breeds is as follows: Pinyang donkey (4 samples), Guangling donkey (4 samples), Hetian donkey (4 samples), Jami donkey (5 samples), Kulun donkey (4 samples), Qingyang donkey (4 samples), Turfan donkey (5 samples), Xinjiang donkey (4 samples), and Yunnan donkey (3 samples). The sequencing was conducted using the high-throughput Illumina HiSeq 4000 technology, with sequencing depths ranging from 7x to 50x (PRJNA431818) (Wang et al., 2020).

**FIGURE 1:**
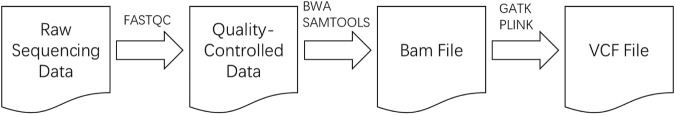
Cluster analysis workflow.

After obtaining the raw donkey genomic data, cluster analysis was conducted to extract SNPs. By using standard donkey genome data as a reference and combining it with several mainstream bioinformatics software tools, the raw genomic data was processed to generate the SNPs dataset. The workflow of the cluster analysis is illustrated in Figure 1.

Initially, the FASTQC software was used for preliminary quality control of the raw donkey sequencing data, removing low-quality segments to ensure the overall data quality, thus improving its reliability (Andrews, 2010). Subsequently, BWA software was employed to align the quality-controlled data with the standard donkey genome, and SAMTOOLS was used to extract sequences that aligned with the reference genome (Li, 2013).

Next, GATK’s MarkDuplicates module was utilized to remove duplicate sequences, followed by the HaplotypeCaller module to generate intermediate files necessary for subsequent analysis (McKenna et al., 2010). These intermediate files were then merged using GATK’s CombineGVCFs module in preparation for the next step. The GenotypeGVCFs module in GATK was used to generate raw SNPs files, which were further filtered and extracted using BCFTOOLS and GATK according to optimal standard conditions.

Finally, PLINK software was employed to apply quality control filtering to the SNPs based on optimal standard conditions, and to remove SNPs with linkage disequilibrium. After using PLINK to remove linkage disequilibrium, the data volume was significantly reduced, ultimately generating a file recording the SNPs data (Purcell et al., 2007).

In summary, the raw donkey sequencing data was first processed using FASTQC for quality control, removing low-quality segments to ensure reliable data. Next, BWA software was used to align the data with the reference donkey genome, followed by the extraction of aligned sequences using SAMTOOLS. To eliminate duplicates, GATK’s MarkDuplicates module was applied, and intermediate files were created using the HaplotypeCaller module. These were then merged and processed using GATK’s CombineGVCFs and GenotypeGVCFs modules to generate raw SNPs data. The SNPs were filtered and further processed with BCFTOOLS and GATK according to standard conditions. Finally, PLINK software was used to apply quality control filtering to remove SNPs with linkage disequilibrium, resulting in a cleaned dataset, which was then saved as a CSV file for further analysis.

In the SNPs dataset derived from the cluster analysis, the SNPs detected across all breeds were uniformly compared. This comparison involved the reference genome’s corresponding bases and the research subjects’ corresponding bases, such as [A/G … G/C, G/T]. During comparison, 0/0 and 1/1 denote homozygous sites and homozygous mutations, while 0/1 represents heterozygous mutations. To integrate with machine learning algorithms, 0/0, 0/1, and 1/1 were encoded as 0, 1, and 2, respectively.

The “curse of dimensionality” in SNPs data presents challenges for machine learning, increasing the time cost of training and predicting classification models. During the exploration, this study attempted to test the method of splitting SNPs data by chromosomes (Altman and Krzywinski, 2018; Venkat, 2018). Given that donkeys have 30 pairs of autosomes and one pair of sex chromosomes, and considering the high interference of sex chromosomes, the study focused on the 30 autosomes. This partitioning resulted in 30 separate datasets, significantly reducing the dimensionality of the SNPs data. Each dataset was used independently to train the classification models, and the classification accuracy of each chromosome was recorded. When new data requires prediction, only the chromosome data with higher classification accuracy is used to predict its category. This improvement significantly enhanced the algorithm’s efficiency. After partitioning the data by chromosomes, each dataset was saved separately and stored in. csv format. When reading the data, all SNPs were treated as feature vectors (X) and all classes as labels (y).

#### 2.5.2 Data partitioning and processing

Due to the insufficient and uneven distribution of sample numbers, coupled with the vastness of animal genome data, class imbalance was exacerbated, posing a significant challenge to constructing machine learning classification models. This imbalance can cause the classification model to favor the classes with larger sample sizes, severely affecting the accuracy of donkey breed prediction and reducing the model’s reliability. To address the issue of insufficient sample numbers, this study employed the Synthetic Minority Over-sampling Technique (SMOTE) (Chawla et al., 2002), which balances the dataset by synthesizing some artificial data points. Previous studies have demonstrated the feasibility of applying SMOTE to biological genomic data. For instance, Reel et al. (2021) mentioned in a review of machine learning and biological data that the SMOTE method can be used to overcome imbalance issues (Reel et al., 2021). Whalen et al. (2022) noted that few real-world datasets are completely balanced, and SMOTE is well-suited for addressing extreme imbalances in genomic data (Whalen et al., 2022).

When applying SMOTE, this study adjusted SMOTE’s parameters by setting k_neighbors to 1, so that each minority class sample was only combined with its nearest neighbor sample to generate new synthetic samples, thereby reducing the complexity of generating synthetic samples and making the generated samples more reflective of the original data. Each class sample was expanded to 100 samples, ensuring that each class was balanced during the expansion process, thus addressing the imbalance in sample data. Subsequently, the dataset was standardized to ensure that each feature contributed equally during the analysis or modeling process.

#### 2.5.3 Machine learning model construction

After data processing, three algorithms—Support Vector Machine (SVM), K-Nearest Neighbors (KNN), and Random Forest (RF)—were directly applied using the Scikit-learn library in Python. After tuning the parameters of these algorithms, the prepared training data was input into the models for training. Table 2 lists the parameters used in the models.

**TABLE 2:** Model parameters and descriptions.

Model	Parameter	Description
Support Vector Machine (SVM)	C = 10.0	C: Penalty parameter value, controlling the trade-off between achieving a low error on the training data and minimizing model complexity.
	kernel = “rbf”	Kernel: Specifies the type of kernel function to be used; ‘rbf’ refers to the Radial Basis Function, ideal for non-linear problems.
	gamma = “auto”	Gamma: Coefficient for the kernel function, with “auto” selecting it based on the inverse of the number of features.
	decision_function_shape = 'ovr'	Decision Function Shape: Strategy for handling multi-class classification problems, using the One-Vs-Rest (OvR) method.
K-Nearest Neighbors (KNN)	Default Configuration	Default parameters are used as tuning showed minimal impact on accuracy.
Random Forest (RF)	n_jobs = −1	n_jobs: Utilizes all available CPU cores for parallel processing, speeding up the training process.
	bootstrap = True	Bootstrap: Enables sampling with replacement from the dataset to construct trees, enhancing the model’s generalization ability.
	oob_score = True	OOB Score: Uses out-of-bag samples to evaluate the model’s performance, particularly useful when data is limited.

For the SVM model, the parameter C represents the penalty parameter, controlling the degree of punishment for classification errors. A larger C value indicates stricter punishment, which can lead to a more complex model that may be prone to overfitting. The Kernel parameter specifies the type of kernel function, with the Radial Basis Function (RBF) kernel selected here, as it is well-suited for nonlinear problems. The Gamma parameter controls the coefficient of the kernel function, with “auto” set to automatically select it, defaulting to the inverse of the number of features. The decision_function_shape parameter determines the strategy for multi-class classification problems, with the One-Vs-Rest strategy employed here. The random_state parameter is set to ensure the reproducibility of the results.

For the KNN algorithm, after tuning the parameters, it was found that accuracy differences were minimal, so the default system configuration was used, with the number of neighbors *K*set to 5 by default.

In the RF algorithm, random_state is also set to ensure the reproducibility of results. The n_jobs parameter is configured to utilize all available CPU cores for parallel processing, thereby accelerating the model’s training speed. The Bootstrap parameter enables bootstrapping, which involves sampling with replacement from the dataset to construct trees, helping to increase the model’s generalization ability. The oob_score (out-of-bag score) is used to estimate the model’s generalization ability effectively, particularly when data is limited, as the OOB evaluation can save data that would otherwise be required for validation.

#### 2.5.4 Model performance evaluation

After training the classification models, they were evaluated using the test data. Performance metrics such as Accuracy, Precision, Recall, and F1 Score were calculated using the data from the confusion matrix to assess the effectiveness of the models.

## 3 Results

Table 3 presents the performance evaluation results of the three best-performing classification algorithms on three specific chromosomes. These results further validate the differences in algorithm performance across different chromosomes and provide critical insights for identifying and optimizing the most suitable classifiers for specific SNPs datasets. This evaluation not only aids in understanding the effectiveness of different algorithms in various genomic regions but also offers guidance for subsequent model improvement and practical application.

**TABLE 3:** Performance evaluation of nine-class classification.

Classifier	Chromosome	Accuracy	Precision	Recall	F1-score
KNN	Chr2	66.66%	76.11%	66.66%	63.83%
SVM	Chr19	51.85%	51.91%	51.85%	46.98%
RF	Chr19	48.14%	40.18%	48.14%	43.51%

The data summarized above shows the performance of three different classifiers—Support Vector Machine (SVM), K-Nearest Neighbors (KNN), and Random Forest (RF)—on different chromosomes. The results indicate significant variations in performance across different chromosomes. Specifically, the KNN classifier performed best on chromosome Chr2, achieving an accuracy of 66.66%, a precision of 76.11%, a recall of 66.66%, and an F1-score of 63.83%. The SVM classifier performed moderately on chromosome Chr19, with an accuracy of 51.85%, a precision of 51.91%, a recall of 51.85%, and an F1-score of 46.98%. The RF classifier showed the poorest performance on chromosome Chr19, with an accuracy of only 48.14%, a precision of 40.18%, a recall of 48.14%, and an F1-score of 43.51%. These performance metrics provide valuable references for selecting the most suitable classifier for specific datasets and tasks.

However, the overall accuracy of the nine-class classification task is relatively low. Despite using the Leave-One-Out Cross-Validation (LOOCV) technique to test all samples, the SNPs data on certain chromosomes performed poorly across all three classifiers, contributing almost nothing to the classification task. Therefore, it is recommended to remove these low-quality data in subsequent training to improve the overall performance of the model. This also demonstrates that the method has a certain level of quality control capability.

Several factors may explain these results. Firstly, although the SNP data derived from cluster analysis underwent quality control, the sequencing depth of the data used in this study was relatively low, which may have affected the overall quality of the SNP data and, consequently, the accuracy of the classifiers. Secondly, some samples may have come from hybrid offspring, leading to a reduction in classification accuracy. Finally, errors in data preprocessing, particularly in the interaction between different bioinformatics software, may have also contributed to the decreased classification accuracy.

As shown in Table 4, after evaluating data quality, certain underperforming samples were removed, as they consistently exhibited poor classification performance across all classifiers and contributed minimally to the classification task. Removing these low-quality data significantly improved the overall accuracy of the classifiers. For example, the accuracy of the worst-performing RF classifier on chromosome Chr6 increased from 48.14% to 75%, precision from 40.18% to 79%, recall from 48.14% to 75%, and F1-score from 43.51% to 75%. The KNN classifier on chromosome Chr14 achieved an accuracy of 85%, a precision of 89.33%, a recall of 85%, and an F1-score of 84.25%. The best-performing SVM classifier on chromosome Chr7 further improved its accuracy to 90%, with a precision of 92%, a recall of 90%, and an F1-score of 88.89%. As low-quality data were removed and the dataset was optimized, the accuracy of the classifiers showed a gradual improvement.

**TABLE 4:** Performance evaluation of five-class classification.

Classifier	Chromosome	Accuracy	Precision	Recall	F1-score
RF	Chr6	75.00%	79.00%	75.00%	75.00%
KNN	Chr14	85.00%	89.33%	85.00%	84.25%
SVM	Chr7	90.00%	92.00%	90.00%	88.89%

These results indicate that in SNPs classification tasks, appropriate data preprocessing and quality control can significantly enhance the performance of classifiers, ensuring more accurate classification results. This is of great importance for genomic data analysis and practical applications.

## 4 Discussion

This study explored the precise classification of donkey breeds by combining SNPs data with machine learning algorithms. The core components of the research included data preprocessing, the use of an improved Leave-One-Out Cross-Validation technique, SMOTE to handle data imbalance, and the construction of classification models using Support Vector Machine (SVM), K-Nearest Neighbors (KNN), and Random Forest (RF). These models were then thoroughly evaluated for their performance in classifying donkey breeds.

The results indicate that different chromosomal data significantly influenced the performance of each classifier. For instance, chromosome Chr19 showed favorable performance with both the Random Forest and Support Vector Machine (SVM) models, while K-Nearest Neighbors (KNN) excelled with chromosome Chr2. Further analysis revealed that after removing low-quality data, the SVM model performed best with chromosome Chr6, KNN with chromosome Chr14, and Random Forest with chromosome Chr7. These findings suggest that certain specific chromosomes may play a critical role in the classification of donkey breeds.

Integrating previous research, the role of chromosomal differences and DNA methylation patterns in species-specific traits may be reflected in these results (Chen et al., 2024). Specifically, genetic variations and DNA methylation regions (such as HypoMRs and HyperMRs) on certain chromosomes could affect gene expression, thereby influencing the phenotypic traits of specific breeds. For instance, hypomethylation or hypermethylation in certain chromosomal regions may be closely related to genetic variations in traits such as reproductive capacity, body size, or other physical characteristics. Therefore, the observed differences in classifier performance across chromosomes could be driven by these chromosomal-specific genetic differences and gene regulatory patterns.

From a technical perspective, the performance differences in classifiers may also be influenced by the quality of the data and the representativeness of the data for each chromosome (Dutrow et al., 2022). Certain chromosomes may harbor more informative genetic markers, which better differentiate between donkey breeds. After the removal of low-quality data, certain chromosomes may provide more distinctive genetic features, enabling the SVM, KNN, and Random Forest models to perform more effectively on those chromosomes.

In conclusion, the identification of chromosomal-specific performance reflects not only the complexity of genetics but also underscores the need for more detailed biological explanations. Specifically, understanding how specific regions on chromosomes influence breed classification will benefit from an integrated analysis of genomic data and phenotypic information. Such an approach could provide valuable insights into the underlying genetic mechanisms at play.

However, despite the initial progress made, challenges and limitations remain in the classification of donkey genomic data. Firstly, the relatively low sequencing depth of the donkey genome data directly affected the quality of the SNPs, thereby impacting the accuracy of the classifiers. Low sequencing depth may lead to insufficient capture of some SNPs, increasing the likelihood of errors in the classification model.

In addition, the limited sample size (37 samples across nine breeds) represents a significant limitation of this study. The small sample size reduces the generalizability of the results, as classifiers may overfit to specific patterns within the dataset rather than capturing broader trends across donkey breeds. This limitation is further exacerbated by the imbalanced distribution of samples among breeds, with certain breeds represented by as few as three samples, making it difficult to establish reliable breed-specific patterns. Moreover, the possibility of some samples coming from hybrid breeds adds further complexity to the classification task. The genetic diversity in hybrid samples creates additional challenges for the models to accurately distinguish between different breeds, potentially resulting in unstable classification results.

In terms of data processing, the use of different bioinformatics tools and software may introduce biases, affecting the consistency and reliability of classification results. Specific preprocessing steps, parameter settings, and methodological choices in this study may have significantly influenced the final results, further increasing the complexity of the analysis. Future efforts should aim to address these limitations by obtaining larger, more balanced datasets with higher sequencing depth and by refining preprocessing protocols to ensure greater consistency across analyses.

Future research should focus on improving data quality by obtaining donkey genome data with higher sequencing depth, which will enhance the reliability of SNPs data and reduce errors caused by data noise. Additionally, expanding the sample size, especially covering more donkey breeds, will help improve the generalization ability of the classifiers and better handle the challenges posed by hybrid samples.

Moreover, although this study employed three classical machine learning algorithms—SVM, KNN, and RF—future research could explore more advanced techniques, such as deep learning models or more complex ensemble learning methods, to further enhance classification accuracy and model robustness. Deep learning methods, such as Convolutional Neural Networks (CNNs) or Recurrent Neural Networks (RNNs), may provide new insights into capturing the complex patterns in SNPs data and improve classification outcomes.

There should also be an emphasis on applying feature selection and data cleansing techniques to ensure that critical genetic markers are accurately captured during high-dimensional data processing, thereby improving model interpretability and predictive performance. Furthermore, integrating other genetic markers or phenotypic data with SNPs data could provide a more comprehensive perspective for donkey breed classification.

Overall, this study provides a preliminary framework for donkey breed classification, but further optimization and validation are needed for practical application. With higher quality data, more advanced algorithms, and comprehensive multi-source data integration, donkey breed classification technology will continue to improve, providing more reliable support for the conservation and utilization of donkey genetic resources. This work not only holds significance for the development of donkey breeds but also contributes to broader genomic research and global biodiversity conservation efforts.

## Data Availability

The original contributions presented in the study are publicly available. This data can be found here: https://doi.org/10.6084/m9.figshare.28728953.v1.
